# Supplementary material for: Clustering of Tau fibrils impairs the synaptic composition of α3‐Na+/K+‐ATPase and AMPA receptors
Source: EMBO J. 2019 Jan 10;38(3):e99871. doi: 10.15252/embj.201899871 (PMC6356061; doi:10.15252/embj.201899871)
Supplement: Supplementary file 1 — Appendix [file EMBJ-38-e99871-s001.pdf]

Clustering of Tau Fibrils Impairs the Synaptic Composition of α3-Na+/K+-ATPase and AMPA receptors

Amulya Nidhi Shrivastava[1,2,3], Virginie Redeker[1,3], Laura Pieri[1,3], Luc Bousset[1,3], Marianne Renner[4], Karine Madiona[1,3,] Caroline Mailhes-Hamon[2], Audrey Coens[1], Luc Buée[5], Philippe Hantraye[3], Antoine Triller[2,#] and Ronald Melki[1,3, #]

APPENDIX

Table of Contents:

Item	Title	Page No.
Appendix Methods	Supplementary Material and Methods	1-4
Appendix Figure S1.	Characterization of fragmented Fib-Tau used throughout this study.	5
Appendix Figure S2.	Fib-Tau used in the work	6
Appendix Figure S3.	Distribution of Fib-Tau in different brain regions post hippocampal injection	7
Appendix Figure S4.	Clustering of Fib-Tau on axons and dendrites in primary neuronal cultures	8
Appendix Figure S5.	Fib-Tau clusters are localized at cell surface	9
Appendix Figure S6.	Unambigous mass spectrometric identification of α3-NKA, GluA1, GluA2, GluN1, and GluN2B as partners of Fib-Tau	10
Appendix Figure S7.	Fib-Tau binding to inhibitory primary spinal cord neurons	11
Appendix Figure S8.	Co-localization of exogenous Fib-Tau with Homer, α3-NKA, AMPA and NMDA receptors in vivo	12
Appendix Figure S9.	Assessment of the interaction between GluA2 and Tau by crosslinking/MS, GluA2 peptides	13
Appendix Figure S10.	Assessment of the interaction between GluA2 and Tau by crosslinking/MS, Tau peptides	14

Supplementary Material and Methods

Immunohistochemistry and Immunocytochemistry

Antigen-retrieval protocol was applied to improve the labelling of the antibodies in brain sections. For this, Sodium citrate buffer (10 mM Sodium citrate, 0.05% Tween 20, pH 6.0) was used for 30min at 95°C (water bath). For Homer-antibody, the antigen-retrieval protocol was not necessary as this antibody works well without this step (Shrivastava *et al*, 2015). Following antigen-retrieval, brain-sections were washed in 1X PBS followed by blocking (0.25% gelatin and 0.2% Triton-X-100 in 1X PBS) for 45 min. Sections were then incubated overnight with the appropriate primary antibodies diluted in 0.125% gelatin and 0.2% Triton-X-100 in 1X PBS PBS as shown in the table below. Following 3 washes (20 min each), slices were incubated with secondary antibodies (FITC conjugated; 1:1000, 3 hr). After washing for nearly 2-3h, sections were mounted onto glass slides using Vectashield (Vector Labs). Images were acquired using a Leica confocal TCS SP8 microscope and processed using ImageJ, Metamorph (Molecular Devices) and Matlab. The list of antibodies used is given in the table below:

Antibody (Supplier)	Immunohistochemistry	Immunocytochemistry
Rabbit-Homer (Synaptic System)	1:1000	1:800
Mouse Gephyrin (Synaptic System)	-	1:400
Mouse MAP2 (Millipore)	1:2000	1:1000
Rabbit Tau (Synaptic System)	-	1:1000
Mouse-α3-NKA (XVIF9-G10, Thermo Scientific)	1:2000	1:1200
Mouse PSD95 (K28/43, Antibodies Incorporated)	-	1:800
Rabbit GluA1 (Synaptic System)	1:1000	1:600
Rabbit GluA2 (Synaptic System)	1:1000	1:600
Mouse GluN1 (Antibodies Incorporated)	-	1:100
Mouse GluN2B (Antibodies Incorporated)	1:1000	1:100

Immunocytochemistry was performed as per standard protocols (Goniotaki *et al*, 2017; Shrivastava *et al*, 2015). Methanol fixation/permeabilization was performed for GluA1-2/PSD and GluN1-2B/Homer as this method improved the labelling of excitatory synaptic proteins. For α3-NKA/Homer, Tritox-X (0.2% in 3% BSA-1XPBS, 10min) was used for permeabilzation since α3-NKA antibody has an intracellular epitope (Shrivastava *et al*, 2015; Azarias *et al*, 2013). Images were acquired using Leica Inverted Spinning Disk microscope (DM5000B, Coolsnap HQ2 camera, Cobolt lasers).

Plasmids and Transfections

Transfection was performed using lipofectamine-2000 (Invitrogen). Transfection medium (TM) was composed of 1 mM sodium pyruvate and 2 mM Glutamax-100X in nerobasal

medium. Appropriate amount of plasmid as indicated in the table below** and 2 µl of lipofectamine-2000 reagent were added separately in 50 µl of TM. After 10 min, the two solutions were mixed and left for another 15 min at room temperature. During this period, culture medium from cells was replaced with pre-warmed TM. The culture medium was stored at 37ºC. 100 µl of Lipofectamine-plasmid mix was then added on top of cells. After 30 min, cells were washed with TM and the original culture medium added back.

**Plasmid**	**Source**	****Amount /Transfection**
pCI-SEP-NRI	(Addgene item #23999) (Kopec *et al*, 2006)	0.75µg
pCI-SEP-NR2B	(Addgene item #23998) (Kopec *et al*, 2006)	0.75µg
pCI-SEP-GluR1	(Addgene item #24000) (Kopec *et al*, 2006)	0.5µg
pCI-SEP GluR2(R)	(Addgene item #24001) (Kopec *et al*, 2006)	0.5µg
α3-NKA-Dendra2	SEP replaced with Dendra2 (Azarias *et al*, 2013)	0.5µg
mRFP-Clc (Clathrin)	(Addgene item #14435) (Tagawa *et al*, 2005)	0.4µg

SEP = Super-ecliptic pHluorin

Cell-surface biotinylation

Cell surface biotinylation experiment was performed using Pierce™ Cell Surface Protein Isolation Kit (Thermo Fischer Scientific) as per manufacturer's instructions. Cortical neuronal cultures aged DIV 17-18 were used for these experiments. Two dishes per condition (control of Fib-Tau) were pooled together. The final elution was performed in NuPAGE™ LDS Sample Buffer (4X, Thermo Fischer Scientific). The level of cell surface endogenous NKA was probed by western blotting using rabbit polyclonal α-NKA (H-300) antibody (Santa Cruz Biotechnology Inc. #sc-28800).

Calcium and Sodium Imaging

Ca^{2+} imaging was performed following Fluo-4 (1 µM) labelling of neurons for 5 min at 37ºC in Krebs recording medium (110 mM NaCl, 4 mM KCl, 1.5 mM $CaCl_2$, 1.2 mM $MgSO_4$, 25 mM NaHCO3, 1 mM NaH_2PO_4, 20 mM HEPES, 10 mM Glucose, pH 7.4). After a final wash, cells were allowed to recover for 10 min to allow de-esterification prior to imaging. Images were acquired on an inverted (IX71, Olympus) fluorescence microscope maintained at 37°C. Time-lapse images were acquired at every 5s to minimize photo-toxicity. Glutamate (10µM final concentration) was bath applied. For quantification, regions of interest were selected on cell body and total-fluorescence intensity was determined on background-subtracted images (ImageJ).

Sodium imaging was performed following loading of neurons with Na^+-sensitive cytosolic ANG2 (Asante NaTRIUM Green 2) dye in Krebs buffer as previously described (5 µM for 30 min in the cell culture medium(Shrivastava *et al*, 2015)). After washing, the coverslips were placed at 37°C for 15 min on a hot plate in Krebs buffer. Imaging was performed within a heated chamber with a perfusion system for exchange of solutions. Images were acquired on an inverted Nikon Eclipse Ti microscope. The K^+ free recording solution (0 mM K^+) had the same composition, except that the NaCl and KCl concentrations were 114 mM and 0 mM, respectively. The 0 mM K^+ recording solution was replaced with normal recording solution and recovery to basal level was monitored until a plateau was reached. Na+-extrusion rate was determined by exponential fitting of the initial decay slope.

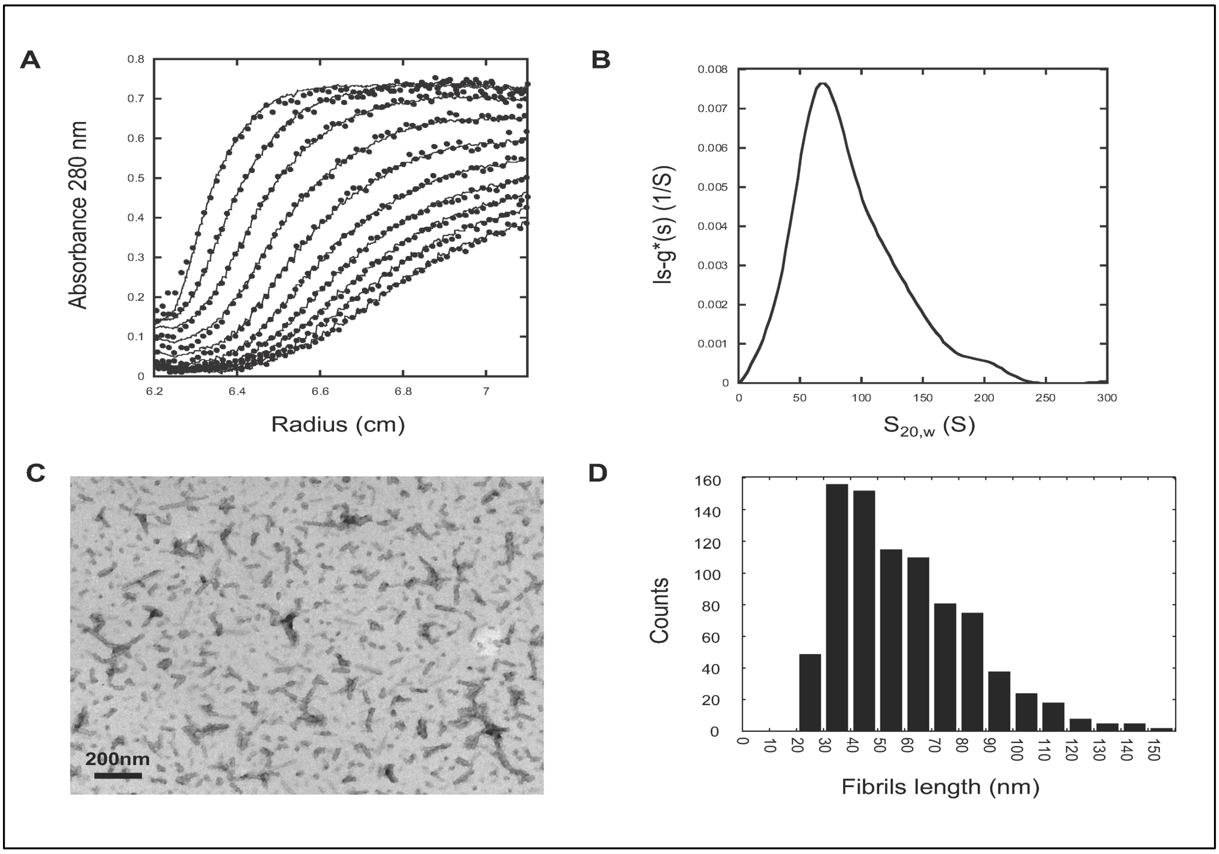

Appendix Figure S1. Characterization of fragmented Fib-Tau used throughout this study. (**A-B**) Sedimentation velocity data for Fib-Tau-1N3R (40 µM monomer concentration) at 15,000 rpm and 20°C in PBS. Shown are sedimentation boundaries obtained at intervals of 5 min (**A**). Sedimentation coefficient g*(s) distribution of Fib-Tau calculated from the sedimentation velocity data of panel A and corrected to s 20,w.

(**C**) Negatively stained quantitative TEM of fragmented Tau-Fib. Scale bar, 200 nm.

(**D**) Length distribution of Fib-Tau obtained by measuring the length of 838 fibrils in 3 different quantitative negatively stained electron micrographs.

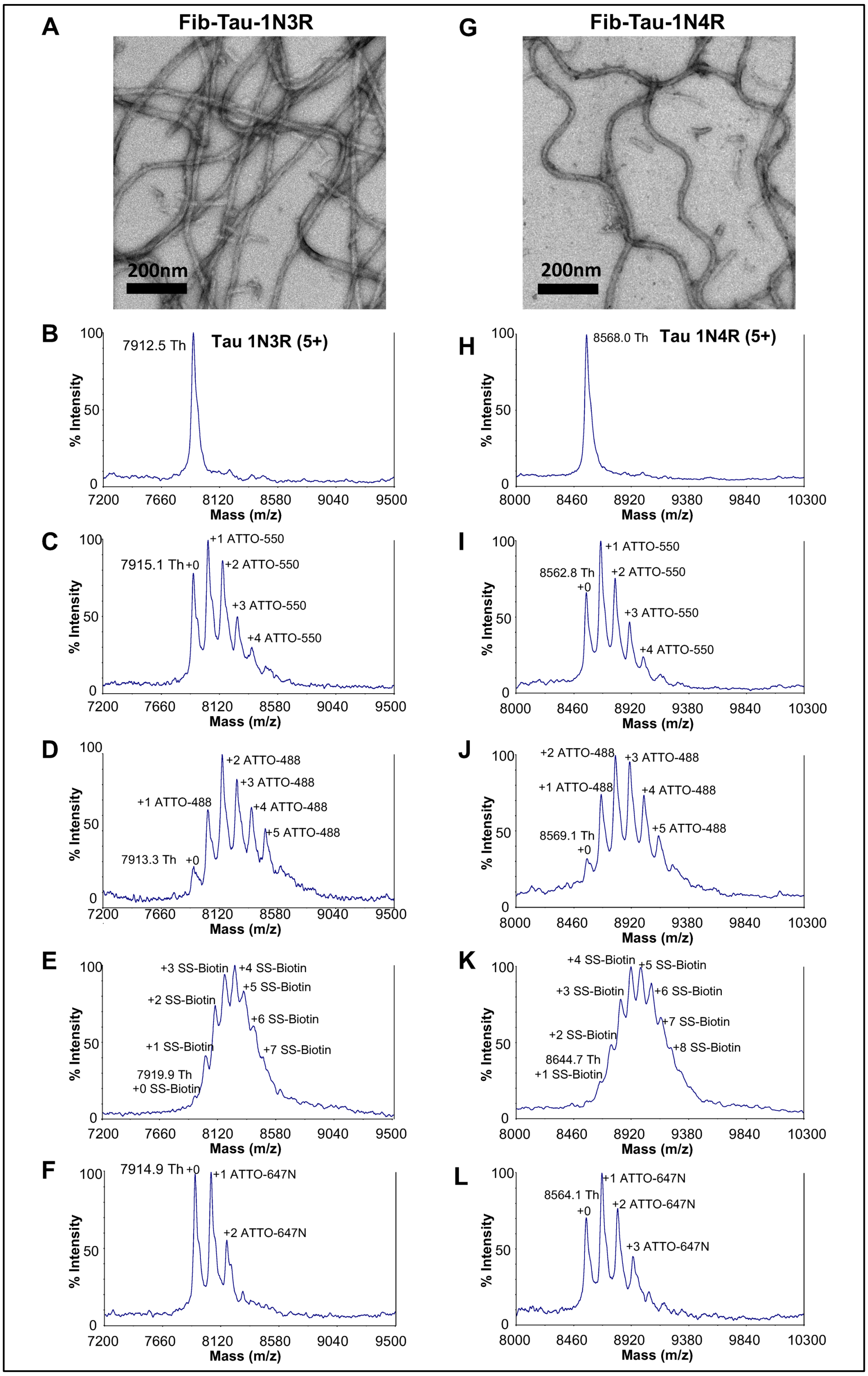

Appendix Figure S2. Fib-Tau used in the work

TEM of negatively stained Fib-Tau-1N3R (**A**) and Fib-Tau-1N4R (**G**) fibrils prior to fragmentation are shown on the top of each column. MALDI-MS spectra from top to bottom of the penta charged ions of unlabeled (**B, H**), ATTO-550-(**C, I**), ATTO-488-(**D, J**), SS-Biotin-(**E, K**) and ATTO-647N-(**F, L**) labeled human Fib-Tau-1N3R and Fib-Tau-1N4R (right) are shown. The m/z value of the first penta charged ion is indicated.

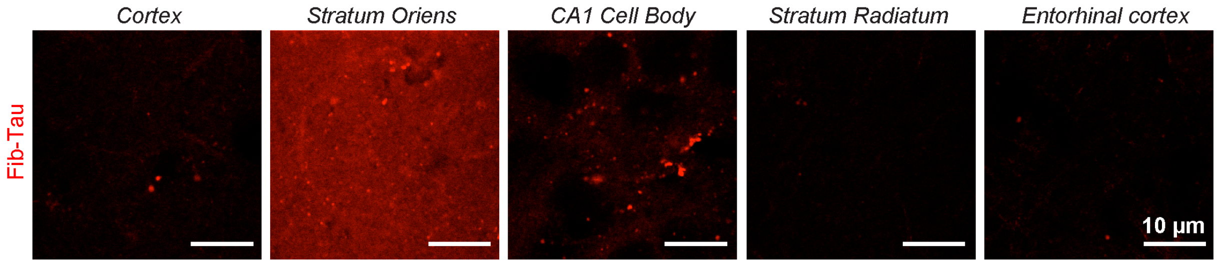

Appendix Figure S3. Distribution of Fib-Tau in different brain regions post hippocampal injection

Distribution of exogenous ATTO 550 labeled Fib-Tau 8 h after injection in the CA1 cell body region (see **Figure 1A**) of the hippocampus. Fib-Tau fluorescence is detected in corpus callosum, Stratum Oriens and CA1 pyramidal cell body layer. Very weak / no fluorescence was detected in regions adjacent to corpus callosum, namely cortex and the entorhinal cortex. No labelling was also detected in the Stratum radiatum. Scale bar, 10µm.

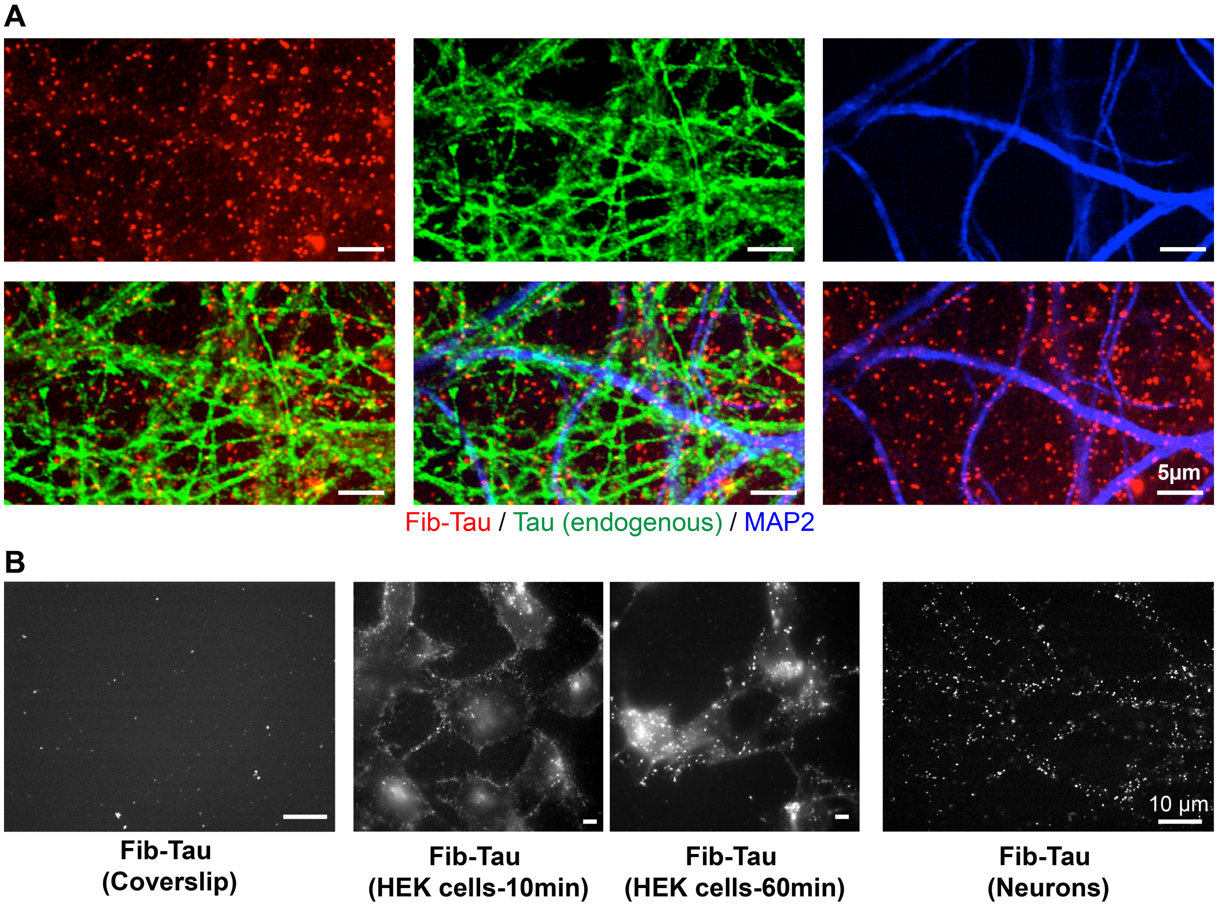

Appendix Figure S4. Clustering of Fib-Tau on axons and dendrites in primary neuronal cultures

(**A**) Clusters of Fib-Tau-1N3R are observed both on axons and dendrites upon exposure of ATTO 550 labeled Fib-Tau (0.36nM, 60min) to mature primary neurons (DIV 21) grown at high density. Scale bar 5µm.

(**B**) Representative images showing Fib-Tau (0.36nM) binding to coverslips without cells (60min exposure), HEK cells (10 or 60 minute exposure) and primary neurons (60 min exposure) grown at low density on the same coverslips. Scale bar 10µm. We did not observe random precipitates.

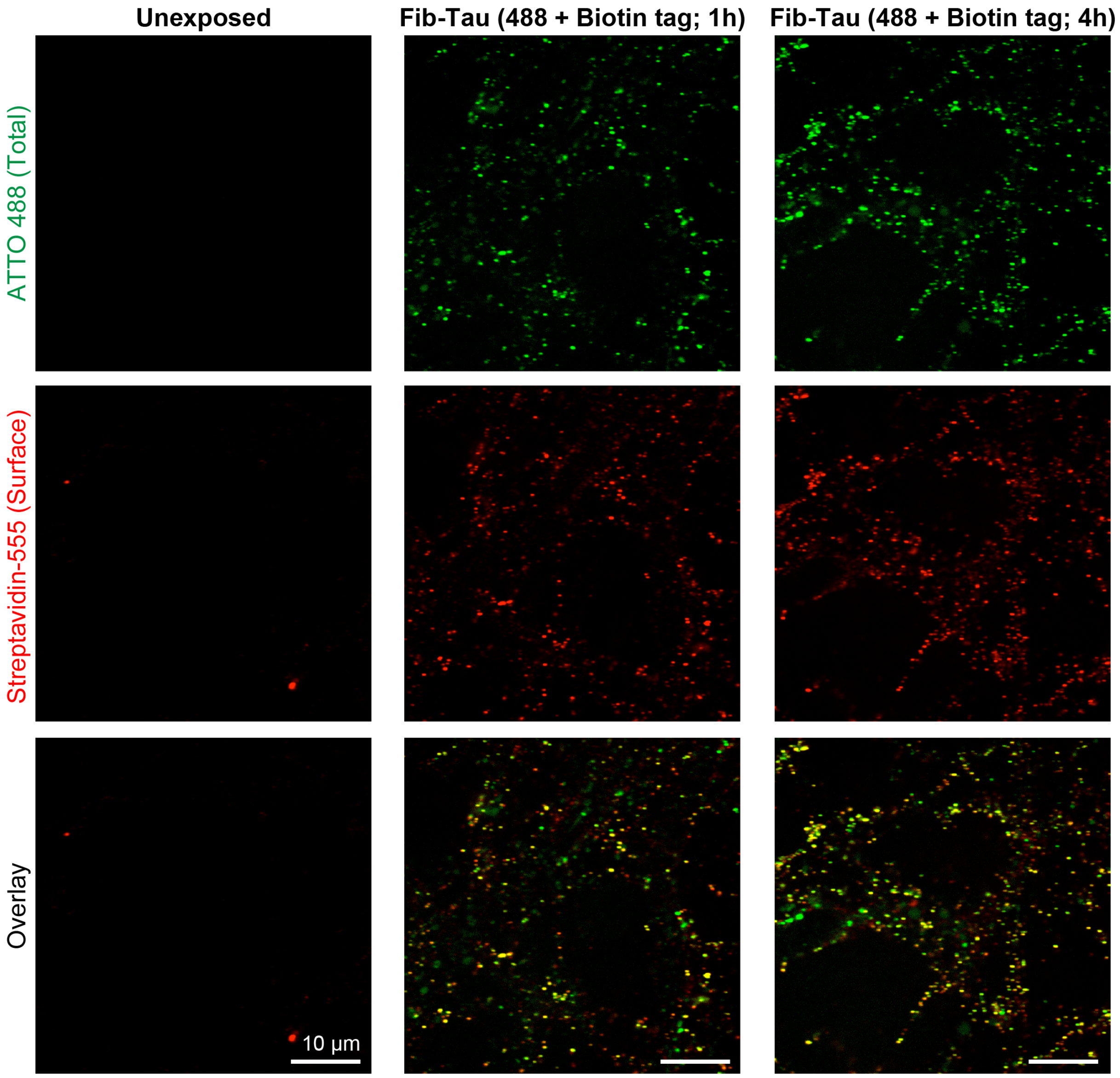

Appendix Figure S5. Fib-Tau clusters are localized at cell surface

Neurons were exposed for 1h or 4h to Fib-Tau 1N3R (0.36nM) labeled both with biotin and ATTO488 (red). Cell surface exposed biotin was labelled using streptavidin-550 (green). Note that most of the clusters of ATTO488 (red) are co-labelled with Streptavidin 550 (green) indicating that the clusters are at the cell surface.

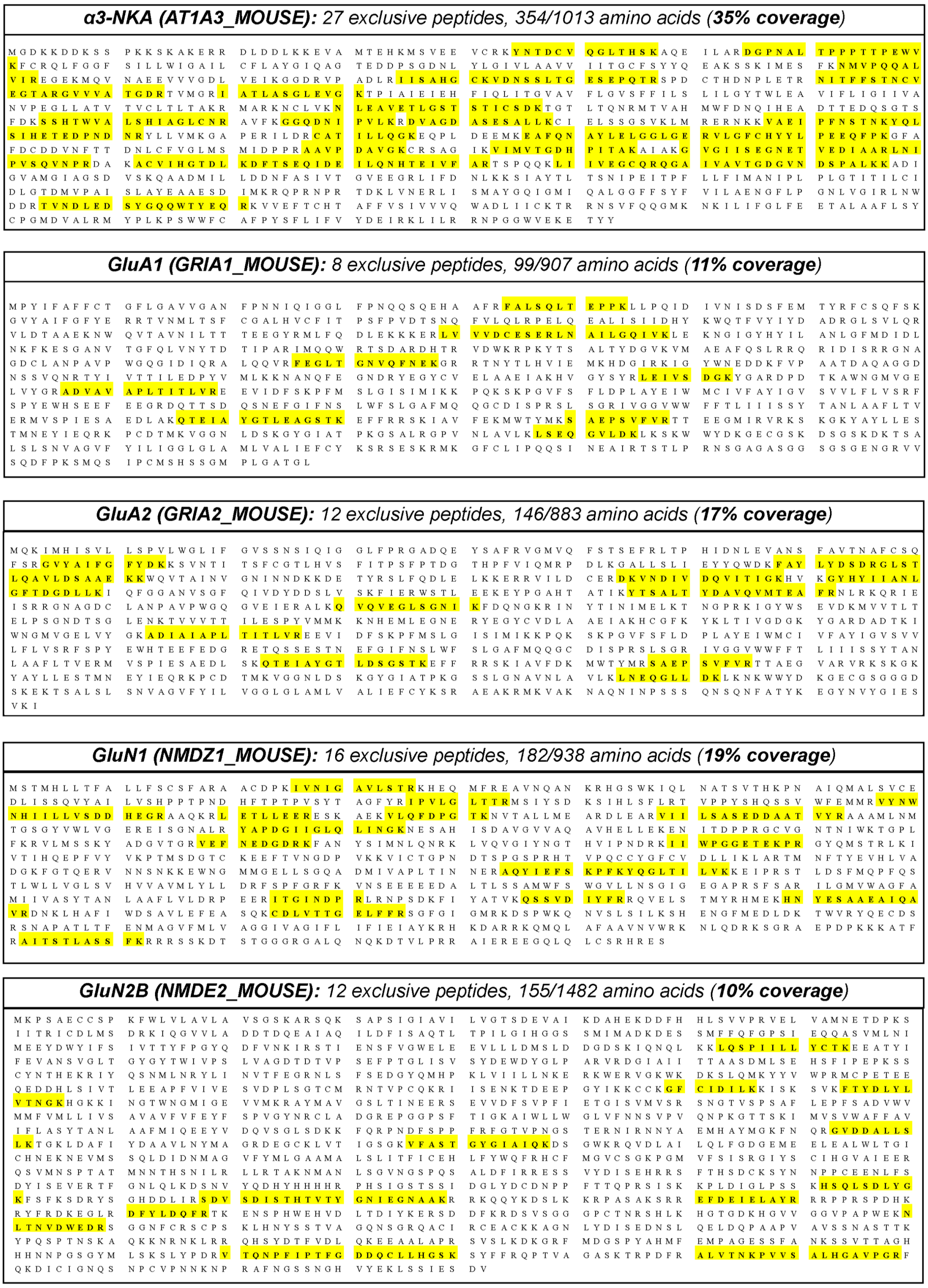

Appendix Figure S6. Unambigous mass spectrometric identification of α3-NKA, GluA1, GluA2, GluN1, and GluN2B as partners of Fib-Tau

Primary structure coverage of α3-NKA, GluA1, GluA2, GluN1, and GluN2B (from top to bottom) obtained following in-gel tryptic digestion of the proteins pulled-down with Fib-Tau-1N3R as described in the material and methods. For each protein, the short name together with the Uniprot accession number, the number of peptides identified by nanoLC-MS/MS analysis of the six replicates, the proportion of protein covered by the unique peptides we identified, are given. The amino acid stretches labeled in yellow correspond to peptides identified by nanoLC-MS/MS.

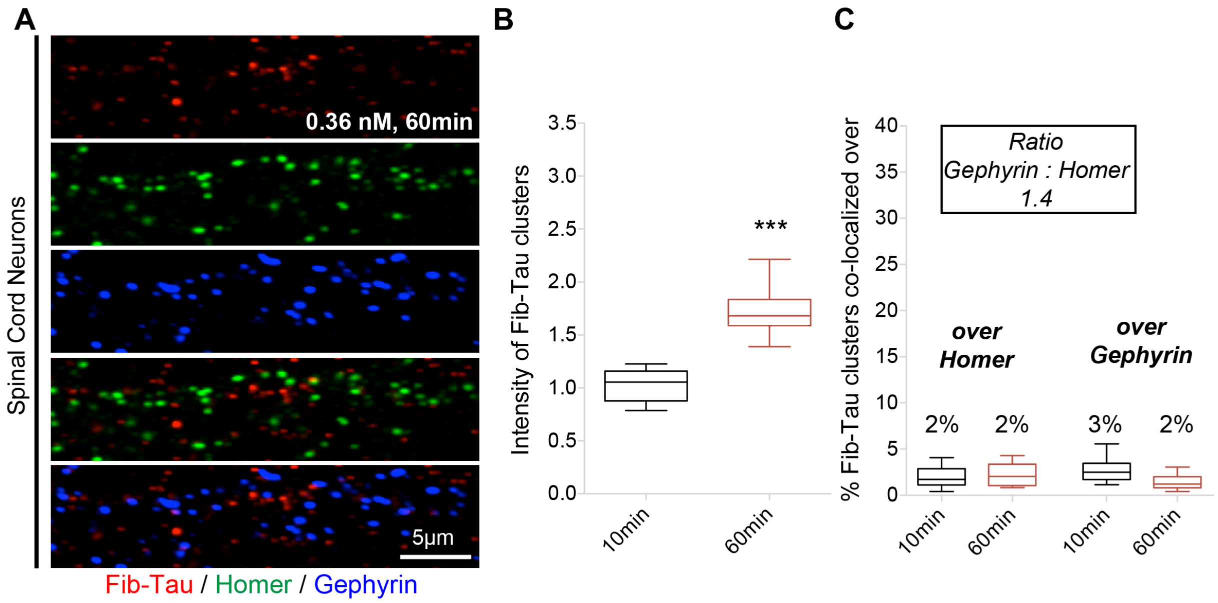

Appendix Figure S7. Fib-Tau binding to inhibitory primary spinal cord neurons

(**A**) Neurons were exposed for 1h to Fib-Tau (0.36nM) labeled both with ATTO550 (red) and synapses immunolabeled for Homer (green) and gephyrin (blue).

(**B**) The intensity of Fib-Tau clusters (size) increased between 10min and 60min exposure.

(**C**) No co-localization of Fib-Tau was detected with synaptic markers. Box plot shows median, inter-quartile range and 10-90% distribution, Mann-Whitney-test, n is number of images analysed, 20 for 10min and 30 for 60min condition. ***p<0.001; ns= not significant.

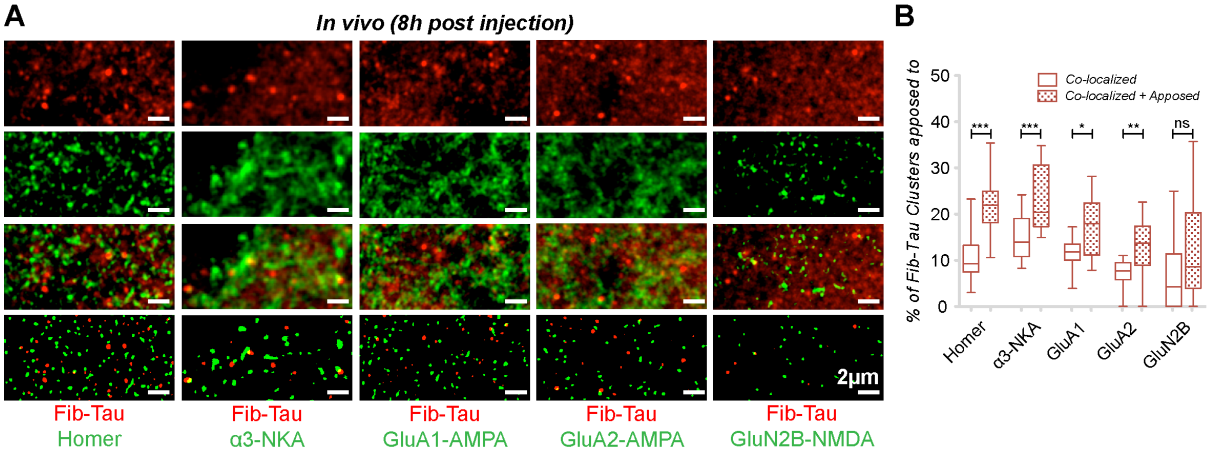

Appendix Figure S8. Co-localization of exogenous Fib-Tau with Homer, α3-NKA, AMPA and NMDA receptors *in vivo*

(**A**) The co-localization of exogenous ATTO 550 labeled Fib-Tau (red) with synaptic proteins (green) was assessed in the stratum radiatum region of the hippocampus (8h after injection of the fibrils, as described in **Figure 1**). Representative images (top three row) are shown following Gaussian filter (radius of 2) to reduce the background for enhanced visualization purpose. Bottom row shows the thresholded images derived from the original images and used for quantitative co-localization analysis.

(B) Quantification of the proportion of Fib-Tau clusters co-localizing with Homer, α3-NKA, GluA1 and GluA2 subunits of AMPA receptors or GluN2B subunit of NMDA receptors (green). *Co-localized*: pixel overlap between red spot and green spot in the thresholded images (**A**, bottom row); *Apposed*: Pixel overlap or red and green pixel adjacent to each other (within 150 nm). Box plot shows median, inter-quartile range and 10-90% distribution, Mann-Whitney-test, n is number of images analysed from 3 experiments (Homer: 15; α3-NKA: 27; GluA1: 15; GluA2: 17; GluN2B: 14). *p<0.05, **p<0.01, ***p<0.001; ns= not significant. Scale bar, 2µm

A

Start-End	Measured Mass	Calculated Mass	Δ ppm	Mascot Ion Score	Modification	Site of modification	Peptide	Exposition (UniProt)
36-45	1142.5026	1142.4992	2.97	52			R.GADQEYSAFR.V	Extracellular
148-156	1148.5161	1148.5138	1.95	42			K.FAYLYDSDR.G	Extracellular
157-171	1501.8013	1501.7988	1.67	91			R.GLSTLQAVLDSAAEK.K	Extracellular
157-172	1629.8957	1629.8937	1.23	61			R.GLSTLQAVLDSAAEKK.W	Extracellular
173-187	1684.8544	1684.8533	0.70	37			K.WQVTAINVGNINNDK.K	Extracellular
194-202	1091.5835	1091.5863	-2.54	46			R.SLFQDLELK.K	Extracellular
194-203	1219.6848	1219.6812	2.95	53			R.SLFQDLELKK.E	Extracellular
194-203	1219.6848	1219.6812	2.95	57			R.SLFQDLELKK.E	Extracellular
207-213	903.4439	903.4484	-4.99	37			R.VILDCER.D	Extracellular
214-228	1655.9152	1655.9094	3.50	96			R.DKVNDIVDQVITIGK.H	Extracellular
216-228	1412.7764	1412.7875	-7.87	76			K.VNDIVDQVITIGK.H	Extracellular
277-283	891.4355	891.4338	1.96	41			R.WSTLEEK.E	Extracellular
284-294	1186.6080	1186.5982	8.25	41			K.EYPGAHTATIK.Y	Extracellular
325-346	2309.0755	2309.0859	-4.51	65			R.GNAGDCLANPAVPWGQGVEIER.A	Extracellular
350-361	1253.6562	1253.6616	-4.27	44	Q->pyro-E (N-term Q)	Q350	K.QVQVEGLSGNIK.F	Extracellular
350-361	1270.6845	1270.6881	-2.82	63			K.QVQVEGLSGNIK.F	Extracellular
385-394	1223.6200	1223.6186	1.15	36			R.KIGYWSEVDK.M	Extracellular
386-394	1095.5218	1095.5237	-1.70	42			K.IGYWSEVDK.M	Extracellular
463-470	801.4575	801.4596	-2.69	35			K.LTIVGDGK.Y	Extracellular
493-506	1465.8939	1465.8868	4.83	60			K.ADIAIAPLTITLVR.E	Extracellular
567-581	1927.8030	1927.8013	0.90	96			R.FSPYEWHTEEFEDGR.E	Extracellular
650-662	1420.6616	1420.6755	-9.79	67	Oxidation (M)	M650	R.MVSPIESAEDLSK.Q	Extracellular
663-677	1569.7414	1569.7522	-6.89	98			K.QTEIAYGTLDSGSTK.E	Extracellular
683-690	1051.5348	1051.5372	-2.29	45	DTSSP (K)	K684	R.SKIAVFDK.M	Extracellular
697-705	990.5153	990.5134	1.92	70			R.SAEPSVFVR.T	Extracellular
752-759	805.4338	805.4334	0.46	36			K.GYGIATPK.G	Extracellular

B

```
Glutamate receptor 2 (GRIA2), 26% sequence coverage
1       MQKIMHISVL LSPVLWGLIF GVSSNSIQIG GLFPRGADQE YSAFRVGMVQ
51      FSTSEFRLTP HIDNLEVANS FAVTNAFCSQ FSRGVYAIFG FYDKKSVNTI
101     TSFCGTLHVS FITPSFPTDG THPFVIQMRP DLKGALLSLI EYYQWDKFAY
151     LYDSDRGLST LQAVLDSAAE KKWQVTAINV GNINNDKKDE TYRSLFQDLE
201     LKKERRVILD CERDKVNDIV DQVITIGKHV KGYHYIIANL GFTDGDLLKI
251     QFGGANVSGF QIVDYDDSLV SKFIERWSTL EEKEYPGAHT ATIKYTSALT
301     YDAVQVMTEA FRNLRKQRIE ISRRGNAGDC LANPAVPWGQ GVEIERALKQ
351     VQVEGLSGNI KFDQNGKRIN YTINIMELKT NGPRKIGYWS EVDKMVVTLT
401     ELPSGNDTSG LENKTVVVTT ILESPYVMMK KNHEMLEGNE RYEGYCVDLA
451     AEIAKHCGFK YKLTIVGDGK YGARDADTKI WNGMVGELVY GKADIAIAPL
501     TITLVREEVI DFSKPFMSLG ISIMIKKPQK SKPGVFSFLD PLAYEIWMCI
551     VFAYIGVSVV LFLVSRFSPY EWHTEEFEDG RETQSSESTN EFGIFNSLWF
601     SLGAFMQQGC DISPRSLSGR IVGGVWWFFT LIIISSYTAN LAAFLTVERM
651     VSPIESAEDL SKQTEIAYGT LDSGSTKEFF RRSKIAVFDK MWTYMRSAEP
701     SVFVRTTAEG VARVRKSKGK YAYLLESTMN EYIEQRKPCD TMKVGGNLDS
751     KGYGIATPKG SSLGNAVNLA VLKLNEQGLL DKLKNKWWYD KGECGSGGGD
801     SKEKTSALSL SNVAGVFYIL VGGLGLAMLV ALIEFCYKSR AEAKRMKVAK
851     NAQNINPSSS QNSQNFATYK EGYNVYGIES VKI
```

C

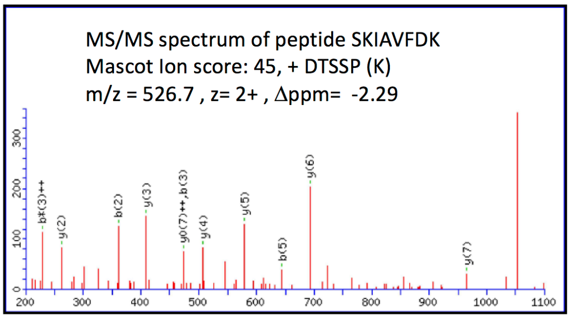

D

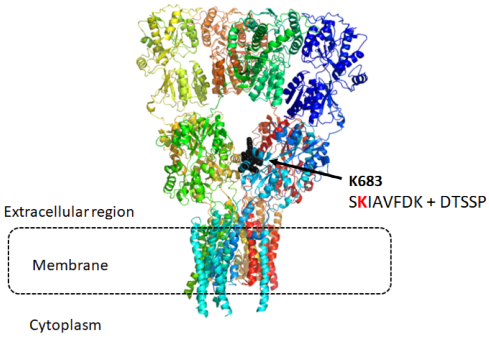

Appendix Figure S9. Assessment of the interaction between GluA2 and Tau by crosslinking/MS, GluA2 peptides

(**A**) List of GluA2 peptides identified from neurons exposed for 10 min to Fib-Tau-1N3R, cross-linked with DTSSP, pulled-down, reduced and alkylated, digested by trypsin and analyzed by MS/MS.

(**B**) Sequence coverage of GluA2.

(**C**) MS/MS spectrum of the GluA2 peptide with one DTSSP cross-linker (+145 Da).

(**D**) Localization of the DTSSP modified K384 residue on the structure of GluA2 adapted from the 5WEK PDB structure.

A

Start-End	Measured Mass	Calculated Mass	Δ ppm	Mascot Ion Score	Modification	Site of modification	Peptide
6-22	2068.8870	2068.8796	3.53	25	Oxidation (M)	M11	R.QEFEVMEDHAGTYGLGDR.K
45-67	2390.0217	2390.0245	-1.19	54			K.ESPLQTPTEDGSEEPGSETSDAK.
68-97	3009.4151	3009.3799	11.7	165			K.STPTAEAEEAGIGDTPSLEDEAAG
142-151	1163.6370	1163.6372	-0.16	49	DTSSP (K)	K145	R.IPAKTPPAPK.T
166-180	1392.6256	1392.6270	-1.00	74			R.SGYSSPGSPGTPGSR.S
183-192	1065.5813	1065.5819	-0.48	57			R.TPSLPTPPTR.E
193-201	1314.6786	1314.6788	-0.11	44	2 DTSSP (K)	K195, K196	R.EPKKVAVVR.T
214-225	1324.7075	1324.7061	1.07	48	Oxidation (M)	M221	R.LQTAPVPMPDLK.N
231-238	860.4619	860.4603	1.81	40			K.IGSTENLK.H
246-257	1387.8083	1387.8075	0.55	85			K.VQIVYKPVDLSK.V
246-257	1532.8264	1532.8272	-0.57	43	DTSSP (K)	K251	K.VQIVYKPVDLSK.V
290-309	2165.0961	2165.0899	2.88	55	DTSSP (K)	K293	R.VQSKIGSLDNITHVPGGGNK.K
294-309	1577.8212	1577.8162	3.17	75			K.IGSLDNITHVPGGGNK.K
326-335	1131.5497	1131.5560	-5.59	56			K.TDHGAEIVYK.S
336-346	1100.5473	1100.5462	1.01	65			K.SPVVSGDTSPR.H

B

```
Tau 1N3R , 48% sequence coverage
1       MAEPRQEFEV MEDHAGTYGL GDRKDQGGYT MHQDQEGDTD AGLKESPLQT
51      PTEDGSEEPG SETSDAKSTP TAEAEEAGIG DTPSLEDEAA GHVTQARMVS
101     KSKDGTGSDD KKAKGADGKT KIATPRGAAP PGQKGQANAT RIPAKTPPAP
151     KTPPSSGEPP KSGDRSGYSS PGSPGTPGSR SRTPSLPTPP TREPKKVAVV
201     RTPPKSPSSA KSRLQTAPVP MPDLKNVKSK IGSTENLKHQ PGGGKVQIVY
251     KPVDLSKVTS KCGSLGNIHH KPGGGQVEVK SEKLDFKDRV QSKIGSLDNI
301     THVPGGGNKK IETHKLTFRE NAKAKTDHGA EIVYKSPVVS GDTSPRHLSN
351     VSSTGSIDMV DSPQLATLAD EVSASLAKQG L
```

C

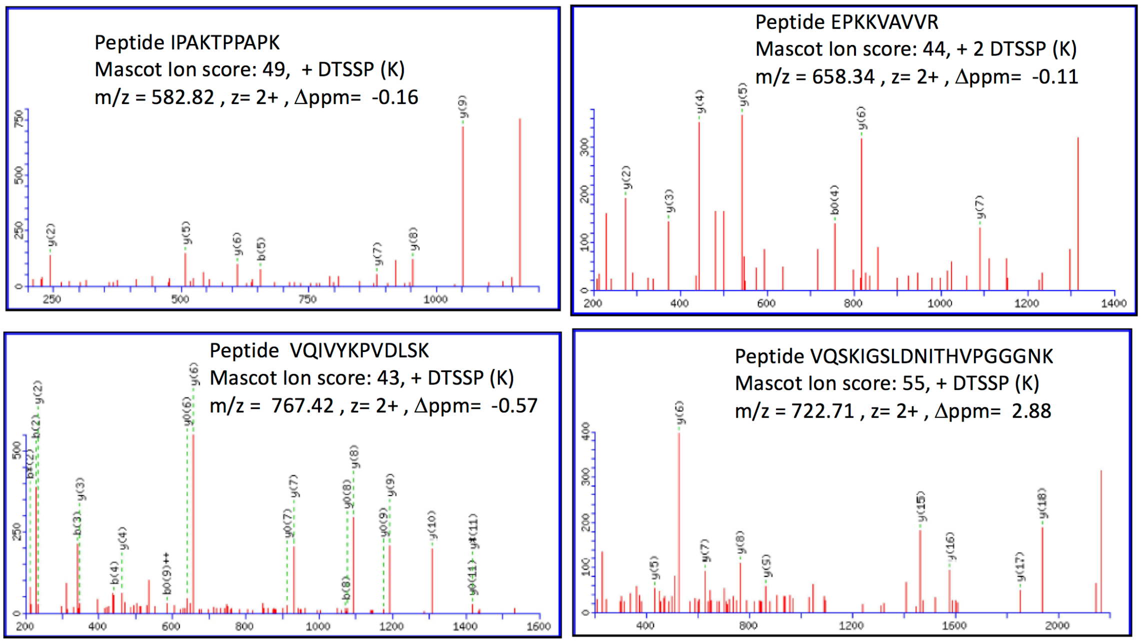

Appendix Figure S10. Assessment of the interaction between GluA2 and Tau by crosslinking/MS, Tau peptides

(**A**) List of Fib-Tau-1N3R peptides identified from neurons exposed for 10 min Fib-Tau-1N3R, cross-linked with DTSSP, pulled-down, reduced and alkylated, digested by trypsin and analyzed by MS/MS

(**B**) Sequence coverage of 1N3R Tau.

(**C**) MS/MS spectra of the four Tau-1N3R peptides with one DTSSP cross-linker (+145 Da).
