# Supplementary material for: Clustering of Tau fibrils impairs the synaptic composition of α3‐Na+/K+‐ATPase and AMPA receptors
Source: EMBO J. 2019 Jan 10;38(3):e99871. doi: 10.15252/embj.201899871 (PMC6356061; doi:10.15252/embj.201899871)
Supplement: Supplementary file 2 — Expanded View Figures PDF [file EMBJ-38-e99871-s002.pdf]

Expanded View Figures

Figure EV1. Identification of intrinsic neuronal membrane proteins that interact specifically with extracellularly applied Fib-Tau-1N4R.

A Venn diagram of 1,065 proteins identified in Fib-Tau-1N4R pull-downs only (red), in control pull-downs only (gray), or in both samples (overlap). Of the 88 proteins identified in both samples, 63 proteins were significantly enriched in Fib-Tau-1N4R pull-downs (*t*-test with *P*-values < 0.05, Benjamini–Hochberg, fold change > 2).

B Distribution of the 379 synaptic and membrane protein interactors of Fib-Tau-1N4R identified in the pull-down experiments. Locations of proteins at the levels of subcellular structures were annotated using the Gene Ontology Cell Component annotation tool of AMIGO 2 (http://amigo.geneontology.org/amigo/landing). Distribution of Fib-Tau interactors in the plasma membrane, pre-synaptic membrane, post-synaptic membrane, pre-synapse, and post-synapse is shown.

C Comparison of synaptic and plasma membrane proteins with extracellular domains significantly enriched in pull-downs from neurons exposed to Fib-Tau 1N4R and 1N3R. For each identified 1N4R protein, the name of the protein, the gene name, the *P*-value (*t*-test with Benjamini–Hochberg correction), and the fold change corresponding to the ratio of spectral counts between exposed neuron and control samples are given.

D Co-immunoprecipitation of exogenous biotin-labeled Fib-Tau-1N4R with α3-NKA, GluA2, and GluN1. α3-NKA, GluA2, and GluN1 were immunoprecipitated using specific antibodies as described in the Materials and Methods section. An 1.8-, 1.2-, and 2.5-fold enrichment in Tau band intensity is observed in α3-NKA, GluA2, and GluN1 immunoprecipitates, respectively, compared to controls performed with pre-immune goat or rabbit IgGs.

E Detection of co-immunoprecipitation by SDS–PAGE and Western blotting. Co-immunoprecipitation of exogenous biotin-labeled Fib-Tau-1N4R with anti-α3-NKA-, GluA2-, and GluN1-specific antibodies. The presence of Fib-Tau in the immunoprecipitate was assessed by probing the nitrocellulose membranes with streptavidin-HRP. Overall, the signal was low with high background. Fib-Tau (**) co-immunoprecipitates with α3-NKA and GluN1-NMDA receptor but not with GluA2-AMPA receptor.

© 2019 The Authors

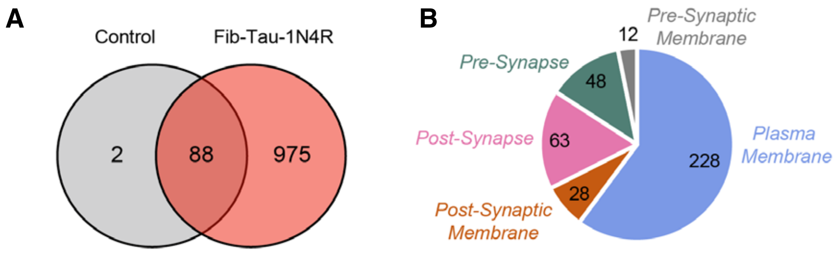

C

Protein Name	Gene Name	p-value (t-test)	Fold Change Tau-1N4R/Control	Identified in Tau-1N3R pulldown
Astrotactin-1	Astn1		-	+
CD 81 antigen	Cd81	<0.00010	∞	+
Excitatory amino acid transporter 1	Slc1a3	<0.00010	∞	+
Glutamate AMPA receptor subunit 1	Gria1		-	+
Glutamate AMPA receptor subunit 2	Gria2	0.00026	∞	+
Glutamate NMDA receptor subunit 1	Grin1	<0.00010	∞	+
Glutamate NMDA receptor subunit 2B	Grin2b	<0.00010	∞	+
Glypican-1	Gpc1	0.002	∞	-
Glypican-2	Gpc2	0.0014	∞	+
Glypican-4	Gpc4	<0.00010	∞	+
Immunoglobulin superfamily member 8	Igsf8	<0.00010	∞	+
Low-density lipoprotein receptor-related protein 4	Lrp4	0.00084	∞	+
Monoacylglycerol lipase ABHD12	Abhd12	0.0003	∞	+
Monoacylglycerol lipase ABHD6	Abhd6	<0.00010	∞	+
Neural cell adhesion molecule 1	Ncam1	<0.00010	∞	+
Neurexin-1 α	Nrxn1	0.002	∞	+
Neurexin-2 α	Nrxn2	<0.00010	∞	+
Neuronal membrane glycoprotein M6-a	Gpm6a	<0.00010	∞	+
Nodal modulator 1	Nomo1	<0.00010	∞	+
Plasma membrane calcium-transporting ATPase 1	Atp2b1	<0.00010	∞	+
Plasma membrane calcium-transporting ATPase 4	Atp2b4	<0.00010	∞	+
Receptor-type tyrosine-protein phosphatase S	Ptprs	<0.00010	∞	+
Renin receptor	Atp6ap2	0.001	∞	-
Na^+/K^+-transporting ATPase subunit α1	Atp1a1	<0.00010	∞	+
Na^+/K^+-transporting ATPase subunit α3	Atp1a3	<0.00010	∞	+
Na^+/K^+-transporting ATPase subunit β1	Atp1b1	<0.00010	∞	+
Thioredoxin-related transmembrane protein 1	Tmx1	0.00015	∞	+
Thioredoxin-related transmembrane protein 2	Tmx2	0.0025	∞	+
Voltage-dependent anion-selective channel protein 1	Vdac1	<0.00010	∞	+
Voltage-dependent anion-selective channel protein 2	Vdac2	<0.00010	9.7	+
Voltage-dependent anion-selective channel protein 3	Vdac3	<0.00010	∞	+

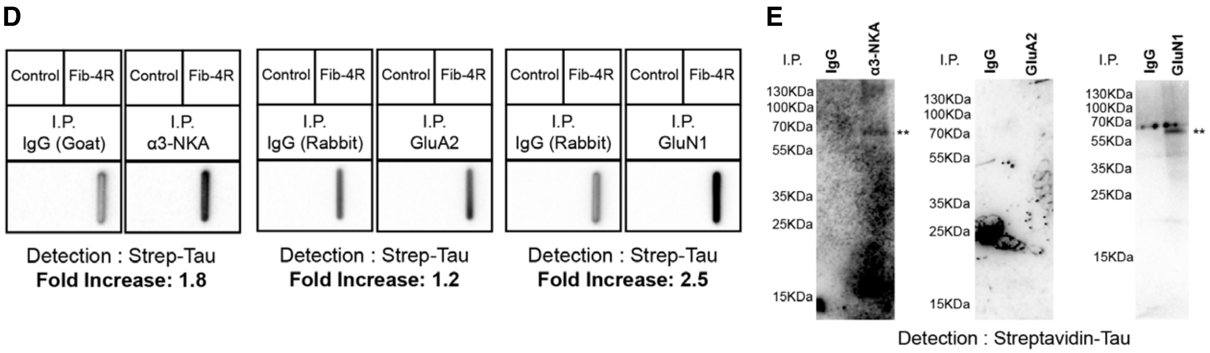

Figure EV1.

 © 2019 The Authors

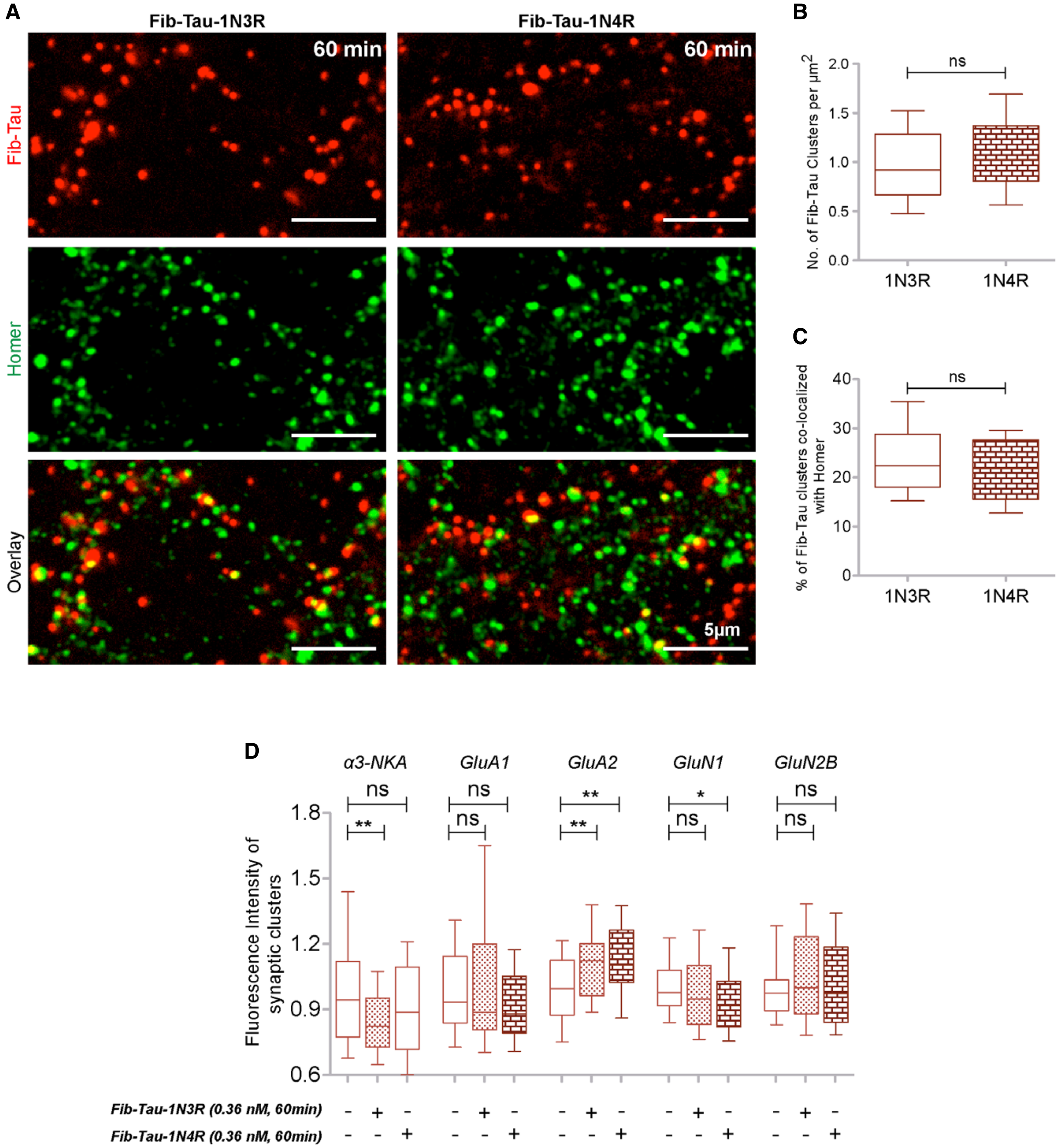

Figure EV2. Fib-Tau-1N4R clusters at excitatory synapses in a manner similar to Fib-Tau-1N3R.

A–C Neurons were exposed for 60 min to ATTO-550-labeled 1N3R or 1N4R Fib-Tau (0.36 nM, red), and excitatory synapses were immuno-labeled (homer, green). A similar clustered distribution/density of Fib-Tau-1N3R and 1N4R is seen (A, top row, B). Fib-Tau isoforms co-localize with homer to similar extents (A, bottom row, C). Box-plot represents median, interquartile range, and 10–90% distribution, Mann–Whitney test, panel (B): n = 37 and 33 images from three experiments, panel (C): n = 27 and 23 images from two experiments. Scale bar, 5 µm.

D Quantification of the fluorescence intensity (size) of synaptic α3-NKA/AMPA/NMDA spots following neurons exposure to Fib-Tau-1N3R or 1N4R (data for 1N3R are also plotted in Fig 5D). A similar reduction in the size of synaptic α3-NKA and increase in the size of synaptic GluA2 subunit containing AMPA receptors clusters were observed for both Fib-Tau-1N3R and 1N4R. Box-plot represents median, interquartile range, and 10–90% distribution, Mann–Whitney test, n is number of images analyzed from 3 to 4 experiments (α3-NKA: 75, 75, 74; GluA1: 95, 95, 55; GluA2: 50, 50, 50; GluN1/N2B: 45, 45, 45). *P < 0.05, **P < 0.01; ns = not significant.

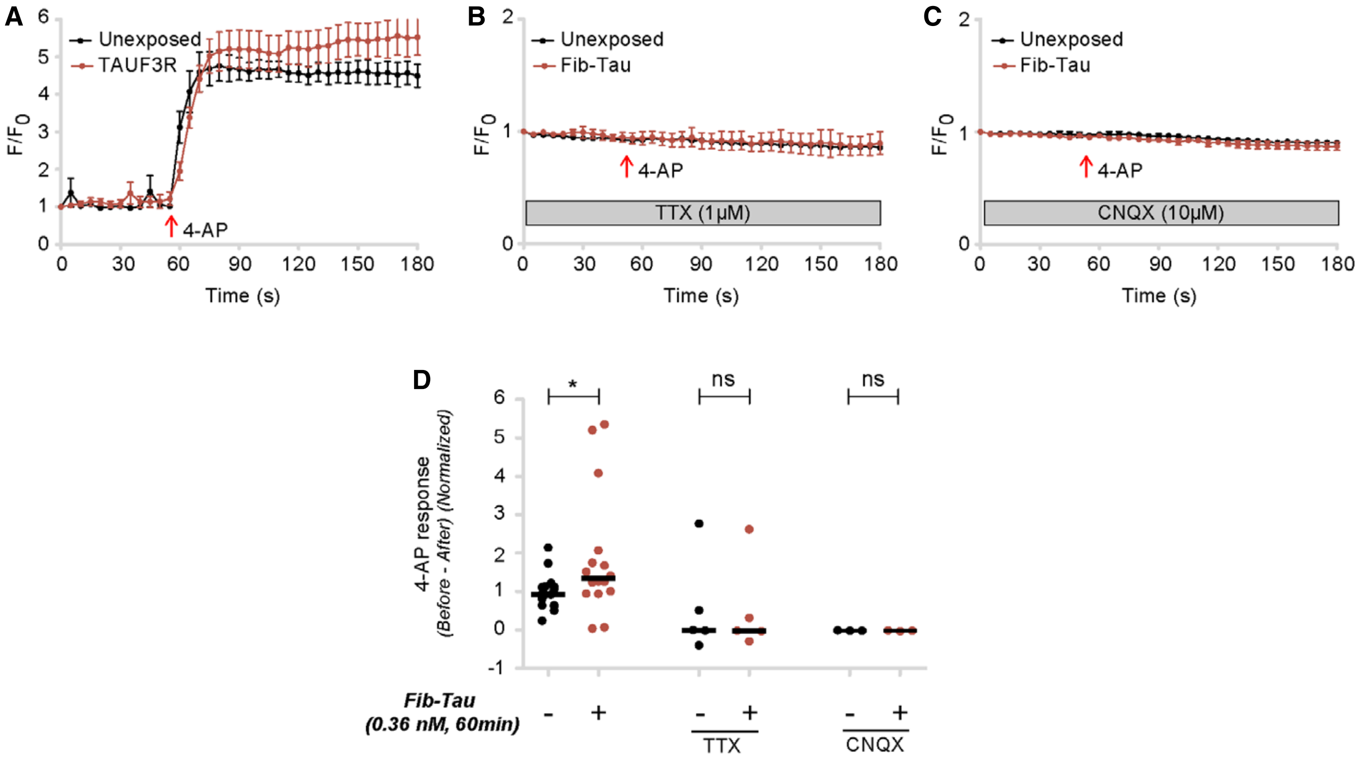

Figure EV3. Enhanced 4-aminopyridine (4-AP)-induced Ca^{2+} response following exposure of neurons to Fib-Tau.

A–D Calcium imaging was performed on neurons unexposed or pre-exposed to Fib-Tau (0.36 nM, 60 min) that were subsequently labeled with Fluo-4 dye. The maximal response to 4-AP (50 μM) was recorded using bath application (red arrow). In normal recording Krebs buffer, neurons exposed to Fib-Tau exhibited slightly higher response than unexposed neurons (A and D). When the buffer contained tetrodotoxin (TTX, 1 μM) to block neuronal activity or AMPA antagonist, CNQX (10 μM), 4-AP failed to elicit a Ca^{2+} response (B–D). Mean ± SEM value of all cells is plotted in panels (A–C); averaged value of all cells per coverslip is plotted in panel (D). Mann–Whitney test, n = number of coverslip (left to right: 16, 16, 5, 5, 3, and 3) from 2 to 5 independent experiments. *P < 0.05, ns = not significant.

Figure EV4. Properties of monomeric Fib-Tau-1N3R.

A Monomeric Fib-Tau is rapidly endocytosed in neurons. Exposure (60 min) of neurons to ATTO-550 (50 nM, red)-labeled monomeric Fib-Tau followed by immunolabeling of homer (green). The majority of monomeric Tau spots were detected intracellularly at this time point.

B–D No change in the diffusion properties of α3-NKA following exposure to monomeric Tau is observed. Single-particle tracking using PALM (SPT-PALM) was performed on α3-NKA-Dendra expressing primary neurons as shown in Fig 7. The averaged diffusion coefficient per cell of α3-NKA was unaltered following exposure to monomeric Tau (50 nM for 60 min) (B). The occupancy time (C) and dwell time (D) of α3-NKA in bound state and in free state were unaltered in neurons exposed to monomeric Tau. Box-plot represents median, interquartile range, and 10–90% distribution, Mann–Whitney test, control: 11 cells and monomeric Tau: 8 cells from two independent experiments.

E–H α-synuclein does not alter monomeric Tau distribution. DIV21 neurons were co-exposed to a defined concentration of monomeric ATTO-550-labeled Tau (50 nM) and increasing concentrations of fibril α-Syn for 60 min (E). Intracellular localization of monomeric Tau and plasma membrane localization of fibrillar α-Syn (E). No alteration in the distribution (F) or fluorescence intensity (G) of monomeric Tau spots was observed upon co-exposure with increasing concentration of fibril α-Syn. Tau and α-Syn spot co-localization was unaffected by increasing concentration of α-Syn (H). Box-plot represents median, interquartile range, and 10–90% distribution, n = 20 images in (G and H) from two independent experiments; one-way ANOVA with Dunnett's *post hoc* test was performed in panel (G).

▶

 © 2019 The Authors

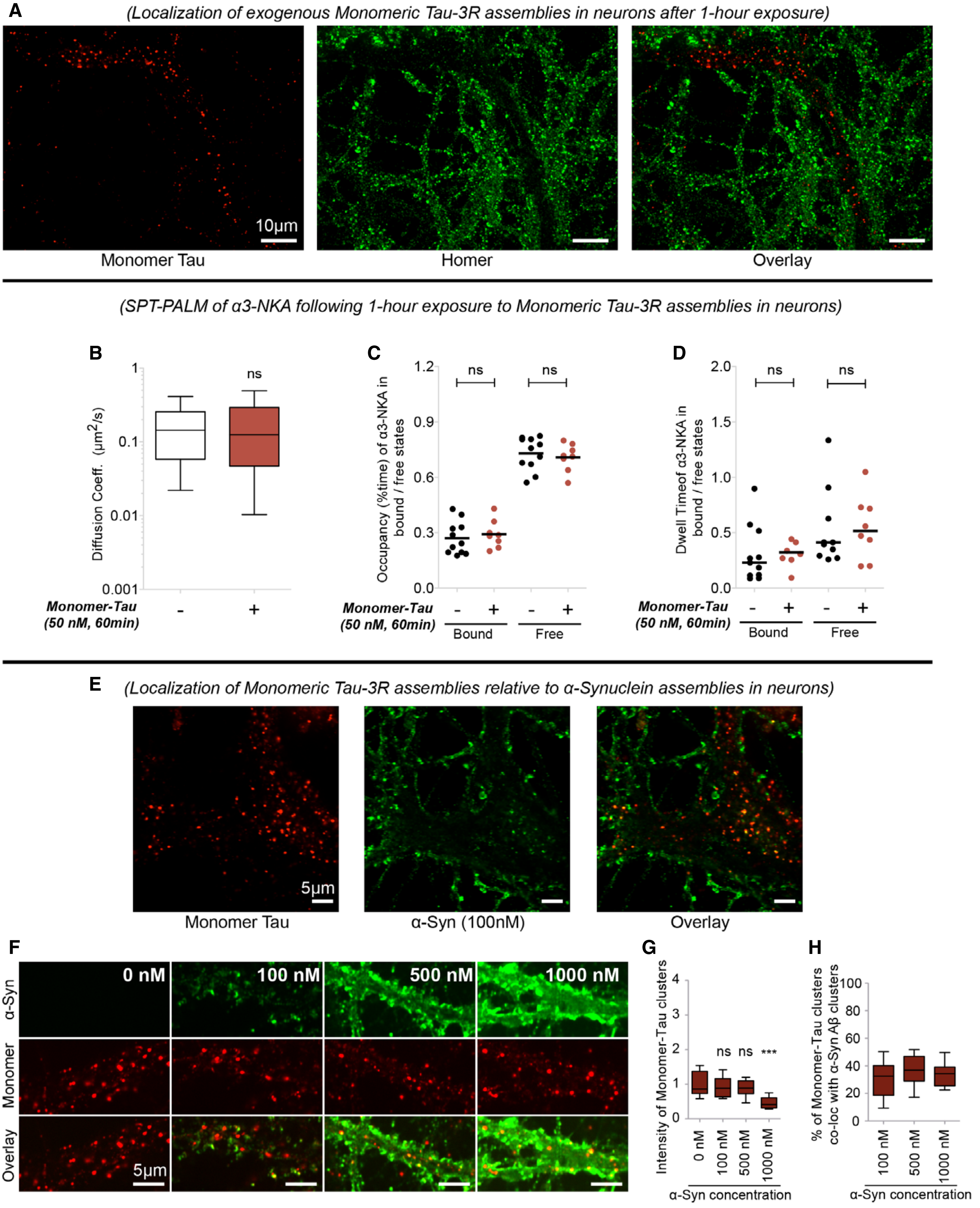
A
(Localization of exogenous Monomeric Tau-3R assemblies in neurons after 1-hour exposure)
10µm
Monomer Tau
Homer
Overlay
(SPT-PALM of α3-NKA following 1-hour exposure to Monomeric Tau-3R assemblies in neurons)
B
Diffusion Coeff. (µm2/s)
Monomer-Tau (50 nM, 60min)
ns
C
Occupancy (%time) of α3-NKA in bound / free states
Bound
Free
D
Dwell Timeof α3-NKA in bound / free states
E
(Localization of Monomeric Tau-3R assemblies relative to α-Synuclein assemblies in neurons)
5µm
α-Syn (100nM)
F
0 nM
100 nM
500 nM
1000 nM
α-Syn
Monomer
Overlay
G
Intensity of Monomer-Tau clusters
α-Syn concentration
***
H
% of Monomer-Tau clusters co-loc with α-Syn Aβ clusters

Figure EV4.

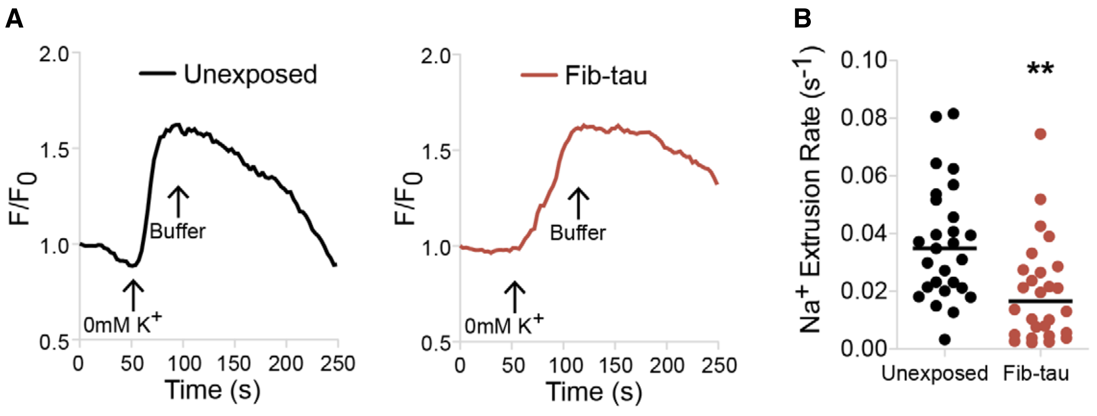

Figure EV5. Compromised α-NKA activity upon exposure of neurons to Fib-Tau.

A, B Sodium imaging was performed on neurons unexposed or pre-exposed to Fib-Tau (0.36 nM, 60′) that were subsequently labeled with ANG-2 dye. Representative traces showing Na^+ dynamics in individual neurons exposed to unlabeled Fib-Tau (A). Following 0 mM K^+ recording solution application, an increase in ANG-2 fluorescence is observed (A). The replacement of 0 mM K^+ recording solution with normal recording solution allows the quantification of Na^+ extrusion rate. Neurons exposed to Fib-Tau exhibit decreased Na^+ pumping rate (B) after changing the 0 mM K^+ recording solution to normal recording solution. Mann–Whitney test, control: 28 cells and Fib-Tau: 27 cells from three experiments, $^{**}P < 0.01$.

 © 2019 The Authors
